# The molecular mechanisms of the bacterial iron sensor IdeR

**DOI:** 10.1042/BST20221539

**Published:** 2023-05-04

**Authors:** Francisco Javier Marcos-Torres, Linda Juniar, Julia J. Griese

**Affiliations:** 1 Department of Cell and Molecular Biology, Uppsala University, 751 24 Uppsala, Sweden; 2 Department of Biotechnology and Environmental Protection, Estación Experimental del Zaidín-CSIC, 18011 Granada, Spain

**Keywords:** allosteric regulation, bacterial transcription regulation, diphtheria toxin repressor, iron homeostasis, iron-dependent regulator, metal-sensing transcription factor

## Abstract

Life came to depend on iron as a cofactor for many essential enzymatic reactions. However, once the atmosphere was oxygenated, iron became both scarce and toxic. Therefore, complex mechanisms have evolved to scavenge iron from an environment in which it is poorly bioavailable, and to tightly regulate intracellular iron contents. In bacteria, this is typically accomplished with the help of one key regulator, an iron-sensing transcription factor. While Gram-negative bacteria and Gram-positive species with low guanine-cytosine (GC) content generally use Fur (ferric uptake regulator) proteins to regulate iron homeostasis, Gram-positive species with high GC content use the functional homolog IdeR (iron-dependent regulator). IdeR controls the expression of iron acquisition and storage genes, repressing the former, and activating the latter in an iron-dependent manner. In bacterial pathogens such as *Corynebacterium diphtheriae* and *Mycobacterium tuberculosis*, IdeR is also involved in virulence, whereas in non-pathogenic species such as *Streptomyces*, it regulates secondary metabolism as well. Although in recent years the focus of research on IdeR has shifted towards drug development, there is much left to learn about the molecular mechanisms of IdeR. Here, we summarize our current understanding of how this important bacterial transcriptional regulator represses and activates transcription, how it is allosterically activated by iron binding, and how it recognizes its DNA target sites, highlighting the open questions that remain to be addressed.

## Introduction

In the early 1990s the groups of Holmes and Murphy identified a transcription factor which represses expression of the diphtheria toxin in *Corynebacterium diphtheriae* in an iron-dependent manner and was, therefore, named DtxR (diphtheria toxin repressor) [1,2]. This protein came to be the founding member of a large family of transcriptional regulators which control metal homeostasis in a wide range of bacteria. While GC-rich Gram-positive bacteria utilize DtxR family transcription factors to regulate both iron (iron-dependent regulator, IdeR) and manganese (manganese transport regulator, MntR) homeostasis, most Gram-negative bacteria and Gram-positive species with low GC content use Fur (ferric uptake regulator) to regulate their intracellular iron status, limiting their use of DtxR regulators to sensing manganese [3–6]. Archaea also utilize more distantly related DtxR homologs to control iron homeostasis [7–9].

The main function of IdeR proteins is to repress the expression of iron uptake genes when intracellular iron levels are sufficient, thereby preventing iron concentrations from reaching toxic levels [10]. When intracellular iron levels are low, metal-free IdeR is inactive, and iron uptake genes are expressed. Once the available and exchangeable ferrous iron concentration is sufficiently high so that iron occupies the metal-binding sites in IdeR, the regulator is activated and binds to its target sites in the genome to prevent transcription.

**Figure 1.**
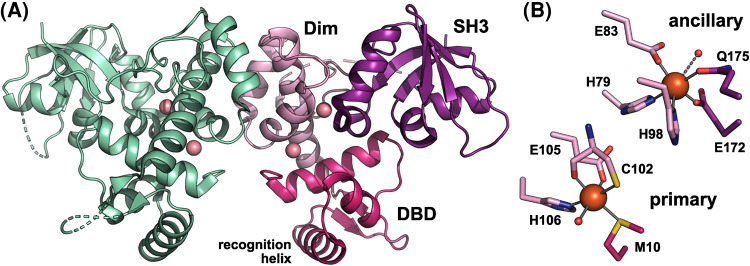
The structure of IdeR in the metallated state. (**A**) The overall structure of the Co^2+^-activated *Se*IdeR dimer (PDB 7B1V) [11], with one subunit colored by domain and the Co^2+^ ions shown as pink spheres (DBD, DNA-binding domain; Dim, dimerization domain; SH3, SH3-like domain). (**B**) Detailed view of the metal-binding sites, showing the Fe^2+^- (and DNA-)bound state of *Se*IdeR (PDB 7B20) [11], using the same coloring scheme as in A (orange spheres, Fe^2+^ ions; small red spheres, water molecules). Cys102 co-ordinates the metal ion with its sulfur as well as its backbone carbonyl oxygen atoms. The water molecule in the ancillary site only weakly co-ordinates the metal ion (3.6 Å distance), as indicated by the dashed line. This figure was prepared with PyMOL (Schrödinger, LLC).

DtxR family proteins consist of an N-terminal DNA-binding domain (DBD) containing a winged helix-turn-helix (wHTH) motif, followed by a helical dimerization domain, and a C-terminal SH3-like domain (Figure 1A) [10,11]. SH3-like domains are structurally similar to eukaryotic SH3 (Src homology 3) domains, which mediate protein–protein interactions in many signaling proteins [12,13], but have no detectable sequence similarity with them.

The best characterized homologs of DtxR proteins are those from *C. diphtheriae* (*Cd*DtxR) and *Mycobacterium tuberculosis* (*Mt*IdeR). More recently, we characterized the IdeR protein from the erythromycin-producing soil bacterium *Saccharopolyspora erythraea* (*Se*IdeR) [11]. In the following we will discuss what we have learned about the molecular mechanisms of IdeR proteins since their discovery 30 years ago.

## The mechanisms of transcription repression and activation by IdeR

IdeR proteins regulate genes involved in the acquisition and storage of iron, such as genes coding for siderophore production and transport, iron transporters, ferritins and bacterioferritins, as well as genes involved in iron-sulfur cluster assembly and genes encoding metabolic enzymes with iron cofactors [11,14–24]. While Fe^2+^-IdeR represses iron uptake, it activates expression of iron storage genes [14,17,19]. Moreover, in *M. tuberculosis* and *C. diphtheriae*, IdeR/DtxR is required not only to control iron homeostasis, but also to regulate virulence [14,15,25]. In the non-pathogenic actinomycete *Streptomyces avermitilis*, IdeR regulates iron metabolism as well as other processes such as secondary metabolism and morphology, thus playing a pleiotropic role in the regulation of the cellular metabolism [18].

DtxR family iron sensors recognize a 19-bp palindrome of four CCTAA repeats (TTAGGTTAGSCTAACCTAA) that is highly conserved across different organisms [11,18,26–28]. In genes repressed by IdeR, this IdeR box overlaps with the transcription start site and/or the −10 region of the promoter. Binding of Fe^2+^-activated IdeR thus blocks access to the promoter for RNA polymerase (RNAP) [15,20]. In contrast, the mechanisms mediating transcriptional activation by IdeR seem to be diverse. Notably, while genes repressed by IdeR generally have only one IdeR box in their promoters, genes which are activated by IdeR tend to have multiple binding sites [15,17]. *Mt*IdeR activates the bacterioferritin and ferritin genes *bfrA* and *bfrB* [14,15,17,19]. Whereas little is known about the mechanism of *bfrA* activation, activation of *bfrB* is mediated by four IdeR boxes upstream of its promoter, one of which partially overlaps the −35 region. IdeR activates this gene in an iron-dependent manner by alleviating the repression exerted by the histone-like protein Lsr2 [17]. Lsr2 binds to the same region as IdeR in an iron-independent manner and distorts the DNA, making the promoter inaccessible for RNAP. IdeR binding (partially) displaces Lsr2 from the promoter region, thus relieving the distortion and activating transcription of the *bfrB* gene. IdeR also contributes to a slight Lsr2-independent induction of *bfrB*, potentially by helping to recruit RNAP [17].

All previously studied IdeR complexes with DNA contained two dimers bound to the 19-bp target sequence [11,27,29–32]. However, *Se*IdeR is also able to recognize what we refer to as a half site, i.e. a 10-bp palindrome consisting of two CCTAA repeats with a 4-bp spacer which binds only a single IdeR dimer. It remains to be investigated if half sites are also found in the genomes of other species utilizing IdeR, and what the function of IdeR is at such sites. Intriguingly, one of the half sites found in *S. erythraea* is in the promoter region of the *nuo* gene cluster coding for the respiratory chain NADH dehydrogenase Nuo complex I, a previously unknown potential target gene of IdeR [11].

## The allosteric activation mechanism of IdeR

### The metal-binding sites and metal selectivity

*In vivo*, IdeR proteins respond to Fe^2+^ [18,33,34]. However, *in vitro* IdeR can be activated by the transition metals Mn^2+^, Fe^2+^, Co^2+^, Ni^2+^, Zn^2+^ or Cd^2+^, although activation by Mn^2+^ and Zn^2+^ is less efficient, while Fe^3+^ and Cu^2+^ are not able to activate IdeR [11,20,26,35–37]. When *Cd*DtxR was expressed in *Bacillus subtilis* (an organism using a Fur protein to sense iron), it responded equally well to Fe^2+^ and Mn^2+^ [38]. These observations illustrate that IdeR is intrinsically no more selective for Fe^2+^ than it needs to be, having evolved to respond to iron within its natural cellular environment in which different metal ions are present in narrowly defined availability ranges [39]. Metal sensors need to bind their target metal ion rapidly and reversibly with a sensitivity that equals the cellular homeostatic setpoint for that metal, which for iron is expected to be in the low micromolar range for most organisms [40–42].

Each IdeR subunit contains two metal-binding sites, named primary and ancillary sites because mutations in the former site have a much more severe effect on IdeR activity than mutations in the latter, although both sites need to be occupied for full IdeR activity [35,43,44] (Figure 1B). To date only one structure of a bacterial IdeR protein activated by its physiological effector Fe^2+^ is available, namely that of Fe^2+^-*Se*IdeR in complex with DNA [11]. However, structures of fully metallated, full-length bacterial DtxR/IdeR proteins show the same metal-binding sites and coordination geometry regardless whether Co^2+^ or Fe^2+^ was used for activation [11,27,29,45]. While most of the metal-binding residues are found in the dimerization domain, the DBD contributes one residue to the primary site, and the SH3-like domain contributes two residues to the ancillary site (Figure 1B). In both sites, the coordination sphere of the metal ion is completed by a water ligand (not visible in all structures, but likely always present). The coordination geometry is almost perfectly octahedral in the primary, but quite distorted in the ancillary site, and the water ligand in the ancillary site is only weakly bound [11].

The ligating residues and coordination geometry make the metal-binding sites suitable, although not exclusively selective for Fe^2+^ [40,46]. Selectivity for Fe^2+^ is increased by the sulfur ligands (Met10 and Cys102) in the primary site, as evidenced by comparison with MntR proteins, in which the corresponding binding site contains an aspartate or asparagine in place of Met10 and a glutamate in place of Cys102. Introducing the IdeR-specific metal ligands to an MntR protein and vice versa switched or broadened the metal responsiveness of the respective variant [38].

Both in *Cd*DtxR and *Mt*IdeR, the ancillary site has significantly higher affinity for most metal ions than the primary site and is consequently occupied first [35,47–50]. Interestingly, IdeR departs from the Irving-Williams series of metal complex stabilities (Mn^2+^ < Fe^2+^ < Ni^2+^ < Co^2+^ < Cu^2+^ > Zn^2+^) [51]. The ancillary site displays the order of relative binding affinities Fe^2+^ ≈ Co^2+^ > Mn^2+^ > Ni^2+^ > Zn^2+^, with an affinity of ∼0.3–0.5 µM for Fe^2+^. In contrast, the primary site exhibits the order of affinities Zn^2+^ ≫ Ni^2+^ > Fe^2+^ > Co^2+^, with an affinity of ∼7–9 µM for Fe^2+^ [47,50]. IdeR only binds one equivalent of Mn^2+^, most likely in the ancillary site, which explains why Mn^2+^ does not efficiently activate IdeR [35,50]. The first equivalent of Zn^2+^ binds to *Mt*IdeR with an affinity of 36 pM, whereas the second equivalent binds with a dissociation constant of 11 µM [50].

Although the assignment of the primary site as the high-affinity site for Zn^2+^ was tentative, if correct, Zn^2+^ would bind to the primary site and Fe^2+^ to the ancillary site of IdeR when both metal ions are present [50]. This study thus implies that *in vivo* IdeR may be activated by binding one equivalent each of Fe^2+^ and Zn^2+^, or by two equivalents of Fe^2+^, allowing IdeR to respond to a wider range of iron levels, which may have particular relevance for pathogenic organisms such as *M. tuberculosis* which encounter severe iron limitation inside their host [15,25]. Based on the metal availabilities in aerobically cultured *Escherichia coli* and the metal affinities stated above, however, the ancillary site would be ∼90% occupied by Fe, while the primary site would be ∼20% occupied by Fe and only ∼4% by Zn [52,53]. Metal availabilities in *M. tuberculosis* may of course be rather different, especially inside macrophages.

Binding of two equivalents of metal ions to IdeR is cooperative [35,47,49,50,54,55]. The primary site metal ligand Glu105 is within hydrogen bonding distance of the ancillary site ligand His79, which has been proposed to be the source of this cooperativity [47]: occupation of the ancillary site leads to ordering of the primary site, allowing binding of the second equivalent of metal. This coupling of the two binding sites may also be the reason why IdeR is more selective for iron than expected based on the Irving–Williams series, in that only Fe^2+^ binding in the ancillary site induces the correct geometry of the primary site.

However, it was also found that cooperative metal binding depends on IdeR dimerization, suggesting that the cooperativity is inter- rather than intramolecular [54]. While the metallated, active state of IdeR forms a stable dimer, *Mt*IdeR and *Cd*DtxR are predominantly monomeric in the apo-form, dimerizing once the first metal ion is bound [35,47,48,50,54,55]. It was, therefore, proposed that activation of IdeR proceeds through a series of steps that includes dimerization.

### Conformational changes in the N-terminal domains

**Figure 2.**
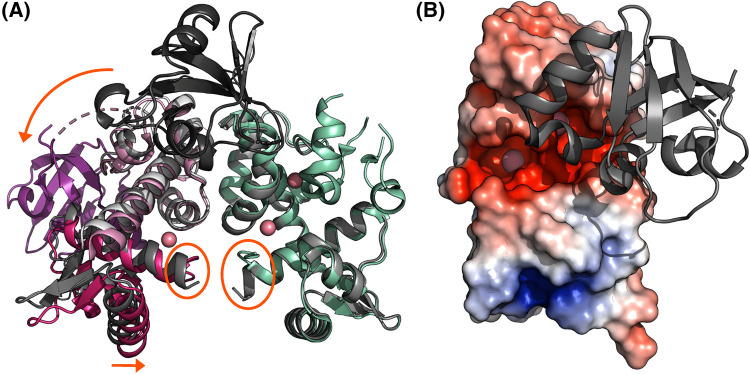
Conformational changes in IdeR upon metallation. (**A**) A superposition of *Cd*DtxR in the metal-free (shades of gray; PDB 1BI2) and the Co^2+^- (and DNA-)bound state (colored as in Figure 1A; PDB 1C0W) [29,57]. Orange ellipses highlight the unwinding of the first turn of the N-terminal helix upon metallation, while orange arrows highlight the caliper-like movement of the N-terminal domains towards each other and the movement of the SH3-like domain, which is only resolved in one subunit of each DtxR dimer. (**B**) The electrostatic surface charge distribution of the DBD and dimerization domain of one of the subunits of the Co^2+^-activated *Se*IdeR dimer (PDB 7B1V) [11] (red, negatively charged; blue, positively charged; white, hydrophobic), with the SH3-like domain shown in cartoon representation (gray). The Co^2+^ ions are shown as pink spheres. Negatively charged funnels attract the metal ions to their binding sites. While the primary site remains solvent accessible in the active state, the SH3-like domain has to detach to allow metal access to the ancillary site. This figure was prepared with PyMOL (Schrödinger, LLC).

As observed in *Cd*DtxR crystal structures, the main difference between apo and metallated states in the N-terminal domains is a slight, caliper-like movement of the DBD of one subunit with respect to the other, bringing the two recognition helices closer to each other and, consequently, to the correct distance to fit into successive major grooves of their DNA target sites [56,57] (Figure 2A). Additionally, the first turn of the N-terminal helix of DtxR unfolds upon metal ion binding to the primary site, to which this helix turn is conformationally coupled [48] (Figure 2A). This conformational change is presumed to be necessary to allow DNA binding, as the intact helix would otherwise clash with the DNA backbone. Circular dichroism (CD) investigations showed that the secondary structure of *Cd*DtxR does not change significantly upon metallation, while its tertiary structure does [35,44]. In contrast, an NMR study observed that the N-terminal domains of IdeR exhibit significant conformational flexibility in the absence of metals, being in a molten-globule like state, and become ordered only upon metal binding, suggesting that changing protein dynamics control activation of IdeR [58]. These results are not in conflict with the crystallographic studies in so far as protein crystallization selects for a stable conformation, and dynamics are inherently invisible to the crystallographic method. Together with Molecular Dynamics simulations, the NMR and CD data suggest that the observed dynamics take place on the level of tertiary rather than secondary structure formation [35,44,58,59].

### The function of the SH3-like domain

The SH3-like domain is connected to the N-terminal domains primarily via the metal-mediated interaction in the ancillary site, which is why it is either not observed or detached from the N-terminal domains in unmetallated IdeR crystal structures [56,57] (Figure 2A). Molecular Dynamics simulations suggest that the SH3-like domain moves toward the DBD upon metal binding and away from it in the absence of metal ions [59], while the structure of metallated IdeR indicates that detachment of the SH3-like domain is required to allow metal access to the ancillary binding site (Figure 2B).

Interestingly, *Mt*IdeR lacking the SH3-like domain forms a stable dimer in the absence of metal ions, implying that in the full-length protein, the SH3-like domain inhibits dimerization until fixed in the active conformation by metal binding to the ancillary site [30]. In analogy to eukaryotic SH3 domains, it has been proposed that the SH3-like domain of *Cd*DtxR interacts with a non-conserved proline-rich region in the loop that connects it to the N-terminal domains [60]. This interaction may stabilize the inactive state, but upon metal binding to the ancillary site the SH3-like domain is released, which in turn allows *Cd*DtxR to adopt its active conformation. In contrast, in *Mycolicibacterium smegmatis* IdeR the SH3-like domains interact in an inter-molecular fashion in the metal-free state, but form the metal-mediated intramolecular interactions with the N-terminal domains upon metallation, thus mediating the metal-dependent activation and inactivation of IdeR in a similar, yet different manner [61]. In *Se*IdeR, a domain swap of the SH3-like domains was observed between adjacent complexes in DNA-bound crystals, which could serve a regulatory function by cross-linking IdeR dimers bound to adjacent binding sites in the genome [11].

It should be noted that while the iron-sensing DtxR family members show a high degree of sequence conservation in their N-terminal domains, they differ substantially in their SH3-like domains. Therefore, it is plausible that the precise function of the SH3-like domain in IdeR activation differs from one homolog to the next, although it does appear to be involved in the allosteric activation mechanism by mediating protein–protein interactions in one way or another.

**Figure 3.**
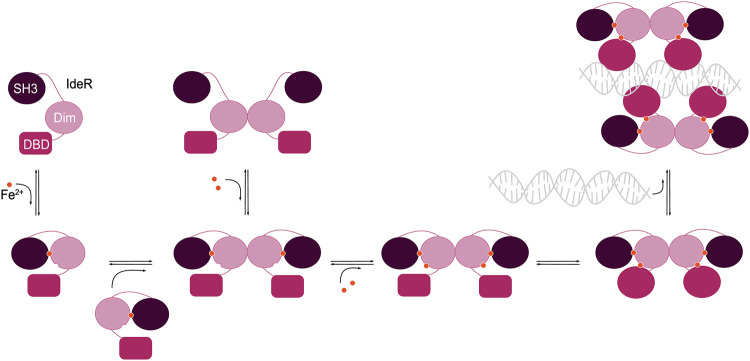
Scheme of the events occurring during allosteric activation of IdeR. Binding of the first iron ion at the ancillary site leads to ordering of the primary site, facilitating binding of the second iron ion there, and promotes dimerization of IdeR. The SH3-like domain moves to interact with the N-terminal domains, and smaller conformational changes and an overall ordering of the N-terminal domains finally lead to the active state of IdeR that can bind to DNA.

To summarize, the following series of events occurs during IdeR activation (Figure 3): the first iron ion binds to the ancillary site, which leads to ordering of the primary site and allows the second iron ion to bind there. Metal binding initiates conformational changes that fix the SH3-like domain in place interacting with the N-terminal domains, promote dimerization, lead to an ordering and rearrangement of the N-terminal domains, and finally result in the active conformation of IdeR which is able to bind to DNA.

## The molecular mechanism of IdeR–DNA recognition

**Figure 4.**
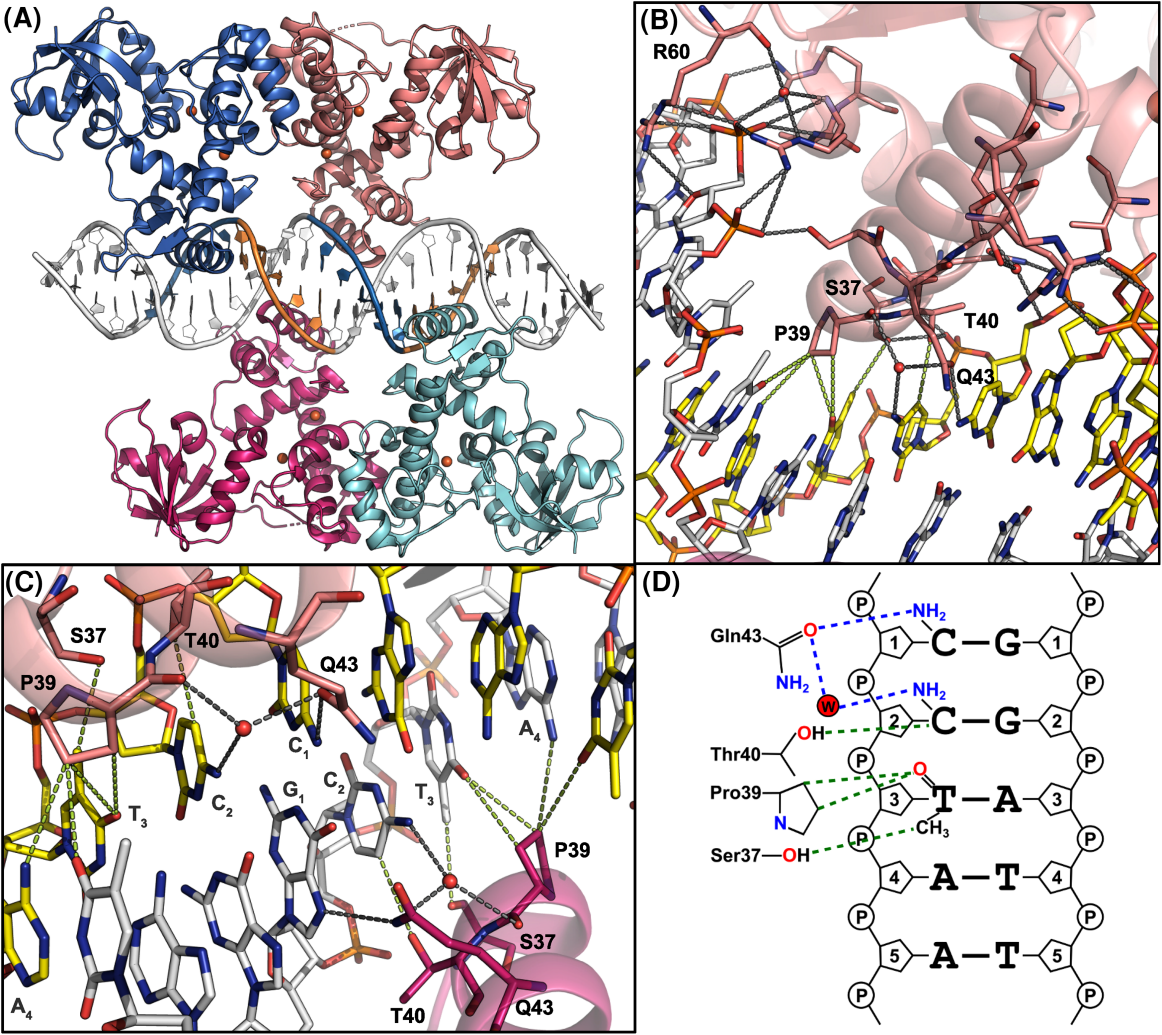
Interactions between IdeR and its DNA recognition sequence. (**A**) Overall structure of Fe^2+^-activated *Se*IdeR in complex with its consensus DNA-binding sequence (PDB 7B20) [11]. IdeR is colored by subunit. The Fe^2+^ ions are shown as orange spheres. The CCTAA repeats in the DNA contacted by each IdeR subunit are shown in blue and orange. (**B**) Interactions between *Se*IdeR and the consensus DNA recognition sequence shown in detail, illustrating how the recognition helix is anchored to the DNA. Only residues mentioned in the text are labeled. (**C**) Interactions between *Se*IdeR and DNA bases, focused on the central G-C basepair in the consensus recognition sequence. In (**B**,**C**), hydrogen bonds and salt bridges are indicated by dashed gray lines, vdW interactions (distances between 3.3 and 3.7 Å) by dashed green lines. (**D**) Two-dimensional representation of the specific interactions between IdeR and DNA bases in the consensus DNA recognition sequence. Hydrogen bonds are shown as dashed blue lines, vdW interactions as dashed green lines. This figure was adapted from [11]. (**A**–**C**) were prepared with PyMOL (Schrödinger, LLC).

Depending on the number of mutations in IdeR recognition sequences compared with the full consensus (TTAGGTTAGSCTAACCTAA), activated IdeR binds to IdeR boxes with an apparent dissociation constant of ∼30–200 nM [11,30,47,50]. Several crystal structures of IdeR homologs in complex with DNA have been reported, namely of those from *C. diphtheriae*, *M. tuberculosis* and *S. erythraea*, bound in most cases to natural promoter sequences, but also to the artificial full consensus [11,27,29–32]. In these structures, two IdeR dimers are bound to the recognition sequence without directly interacting with each other, each subunit contacting one of the four CCTAA repeats of the palindrome (Figure 4A). The DNA in these complexes is in a distorted B-DNA conformation, exhibiting slight bends of the helix axis around each IdeR recognition helix as well as wider and shallower major and minor grooves where the recognition helices are inserted [11,27,29]. In all cases essentially the same interactions between IdeR and the DNA can be observed. In particular the interactions with DNA bases are formed by conserved residues in the recognition helix of the wHTH motif, namely Ser37, Pro39, Thr40 and Gln43 [11] (Figure 4B–D).

The recognition helix of the wHTH motif inserts into the major groove of the DNA target, anchored on both edges of the major groove by hydrogen bonds and salt bridges with the sugar-phosphate backbone of the DNA. These interactions are formed by residues from the first helix of the wHTH motif on one side and residues from the recognition helix on the other. The wing of the wHTH motif contacts the minor groove edge using a conserved arginine (Arg60), thus clamping the backbone between the wing and the first helix of the wHTH motif (Figure 4B). IdeR forms only one direct hydrogen bond with a DNA base, between Gln43 and the first cytosine of each CCTAA repeat (or guanine at the central G-C basepair of the palindrome). A second, water-mediated hydrogen bond is formed between Gln43, the backbone carbonyl group of Pro39, and the second cytosine [11]. Ser37 and Pro39 are in van der Waals (vdW) distance of the thymine in the third position of the repeat. Thr40 is in close proximity to the cytosine in position 2, such that the methyl group of a thymine in this position clashes with the threonine side chain [11] (Figure 4B–D). Finally, the side chain of Pro39 is in vdW distance of the A-T basepair in the fourth position of the CCTAA repeat, but these interactions appear to be unspecific, as the distances do not change significantly if the bases are exchanged [11].

Early studies concluded that these IdeR–base interactions must be responsible for target site recognition, although the paucity of base-specific interactions was noted. Gln43 was observed to interact with different bases in different subunits of the *Mt*IdeR–DNA complex, suggesting that it does not contribute to specificity [27]. While it had been shown that the thymine in position 3 of the CCTAA repeat is important for DtxR recognition [28,32], the question then arose if the vdW interactions with this thymine alone were enough to explain the observed sequence specificity of IdeR. A systematic mutational analysis of *Se*IdeR and its target sequence revealed that these base interactions formed by IdeR are in fact not required for recognition [11]. Instead, IdeR uses a shape readout mechanism to recognize its target sites in the genome, that is, it recognizes sequence-dependent features of the shape and/or flexibility/deformability of the DNA backbone rather than reading the base sequence by directly interacting with the bases [11,62,63]. The binding of two IdeR dimers to target sequences is cooperative [11,32,35]. Given that the dimers do not directly interact with each other, this cooperativity must be mediated by the DNA, with the binding of one IdeR dimer shaping the DNA in such a way as to create a better binding site for the second dimer [11].

So how does IdeR read DNA shape? Analysis of the DNA conformation in the IdeR–DNA complexes, and prediction of the shape features of sequence variations that are and are not recognized by IdeR, suggest that IdeR reads minor groove width and/or deformability [11]. The TpA steps in the target sequence (i.e. the T-A dinucleotides in the CC**TA**A repeats) are likely to be of particular importance for recognition because they make the minor groove easy to deform [62]. Given that mutation of the thymine in position 3 of each repeat abolishes recognition by IdeR, but mutation of the IdeR residues that interact with this base does not, minor groove deformability may be the primary reason why the thymine in this position is important for recognition [11,28,32]. Work is underway in our laboratory to test this hypothesis.

## Open questions

Although in recent years, research into DtxR/IdeR has focused primarily on drug development [64–67], the regulatory mechanisms of these proteins are in fact far from fully understood. The role of DtxR/IdeR has been intensively studied for pathogens, in particular of the genera *Mycobacterium* and *Corynebacterium*. In contrast, the role of IdeR regulation in antibiotic-producing actinomycetes is understudied, despite their importance for antibiotic discovery and production [68,69]. Many questions also remain regarding the molecular mechanisms of allosteric activation and DNA recognition of IdeR. Accurately measuring protein–metal-binding affinities is far from straightforward, resulting in ambiguous interpretations [70]. Moreover, we have only just begun to scratch the surface of the DNA recognition mechanism of IdeR [11]. The most important unresolved questions are:

(i) Which genes does IdeR regulate in free-living, non-pathogenic actinomycetes? Which role does it play in controlling primary and secondary metabolism?
(ii) What is the role of IdeR at half target sites which only bind one IdeR dimer?
(iii) How does IdeR activate certain genes?
(iv) Does IdeR interact with other factors in the cell to accomplish its tasks?
(v) What is the source of the metal-binding cooperativity? Is it intra- or inter-molecular? How does metal binding regulate IdeR dimerization?
(vi) What exactly is the role of the SH3-like domain in activation in different homologs?
(vii) What is the affinity of different IdeR homologs for Fe^2+^ as opposed to physiologically relevant competitors? Does Zn^2+^ play a role in IdeR activation *in vivo* in different organisms?
(viii) Which DNA shape features does IdeR recognize and how? How does the DNA mediate cooperative IdeR binding?

## Perspectives

- IdeR regulates iron homeostasis in many bacterial species of medical and industrial importance. It also controls virulence in bacterial pathogens, making it an interesting drug target, while in non-pathogenic, antibiotic-producing actinomycetes, this transcription factor influences multiple cellular processes, including secondary metabolism.
- IdeR is allosterically activated by binding two iron ions in a process which is mediated by changing protein dynamics and protein–protein interactions. Activated IdeR finds its target sites in the genome using a shape readout mechanism and then represses or activates transcription.
- Although IdeR has been studied for 30 years, its molecular mechanisms remain incompletely understood. A better understanding of these mechanisms will aid both drug development against IdeR itself and the discovery and production of new antibiotics by genetic manipulation of its host organisms.

## Open Access

Open access for this article was enabled by the participation of Uppsala University in an all-inclusive *Read & Publish* agreement with Portland Press and the Biochemical Society under a transformative agreement with Bibsam.

## Abbreviations

CD: circular dichroism
Cd: *Corynebacterium diphtheriae*
DBD: DNA-binding domain
DtxR: diphtheria toxin repressor
Fur: ferric uptake regulator
IdeR: iron-dependent regulator
MntR: manganese transport regulator
Mt: *Mycobacterium tuberculosis*
RNAP: RNA polymerase
Se: *Saccharopolyspora erythraea*
SH3: Src homology 3
vdW: van der Waals
wHTH: winged helix-turn-helix

## References

[1] Boyd, J., Oza, M.N. and Murphy, J.R. (1990) Molecular cloning and DNA sequence analysis of a diphtheria tox iron-dependent regulatory element (dtxR) from *Corynebacterium diphtheriae*. Proc. Natl Acad. Sci. U.S.A. 87, 5968–5972 10.1073/pnas.87.15.5968 2116013 PMC54451

[2] Schmitt, M.P. and Holmes, R.K. (1991) Iron-dependent regulation of diphtheria toxin and siderophore expression by the cloned *Corynebacterium diphtheriae* repressor gene dtxR in *C. diphtheriae* C7 strains. Infect. Immun. 59, 1899–1904 10.1128/iai.59.6.1899-1904.1991 1828057 PMC257940

[3] Hantke, K. (2001) Iron and metal regulation in bacteria. Curr. Opin. Microbiol. 4, 172–177 10.1016/S1369-5274(00)00184-3 11282473

[4] Steingard, C.H. and Helmann, J.D. (2023) Meddling with metal sensors: Fur-family proteins as signaling hubs. J. Bacteriol. 0, e00022-23 10.1128/jb.00022-23 PMC10127796 37010421

[5] Sevilla, E., Bes, M.T., Peleato, M.L. and Fillat, M.F. (2021) Fur-like proteins: beyond the ferric uptake regulator (Fur) paralog. Arch. Biochem. Biophys. 701, 108770 10.1016/j.abb.2021.108770 33524404

[6] Rudolph, G., Hennecke, H. and Fischer, H.-M. (2006) Beyond the Fur paradigm: iron-controlled gene expression in rhizobia. FEMS Microbiol. Rev. 30, 631–648 10.1111/j.1574-6976.2006.00030.x 16774589

[7] Leyn, S.A. and Rodionov, D.A. (2015) Comparative genomics of DtxR family regulons for metal homeostasis in archaea. J. Bacteriol. 197, 451–458 10.1128/JB.02386-14 25404694 PMC4285986

[8] Martinez-Pastor, M., Lancaster, W.A., Tonner, P.D., Adams, M.W.W. and Schmid, A.K. (2017) A transcription network of interlocking positive feedback loops maintains intracellular iron balance in archaea. Nucleic Acids Res. 45, 9990–10001 10.1093/nar/gkx662 28973467 PMC5737653

[9] Yeo, H.K., Park, Y.W. and Lee, J.Y. (2014) Structural analysis and insight into metal-ion activation of the iron-dependent regulator from *Thermoplasma acidophilum*. Acta Crystallogr. D Biol. Crystallogr. 70, 1281–1288 10.1107/S1399004714004118 24816097

[10] Bradley, J.M., Svistunenko, D.A., Wilson, M.T., Hemmings, A.M., Moore, G.R. and Le Brun, N.E. (2020) Bacterial iron detoxification at the molecular level. J. Biol. Chem. 295, 17602–17623 10.1074/jbc.REV120.007746 33454001 PMC7762939

[11] Marcos-Torres, F.J., Maurer, D., Juniar, L. and Griese, J.J. (2021) The bacterial iron sensor IdeR recognizes its DNA targets by indirect readout. Nucleic Acids Res. 49, 10120–10135 10.1093/nar/gkab711 34417623 PMC8464063

[12] Qiu, X., Pohl, E., Holmes, R.K. and Hol, W.G.J. (1996) High-resolution structure of the diphtheria toxin repressor complexed with cobalt and manganese reveals an SH3-like third domain and suggests a possible role of phosphate as co-corepressor. Biochemistry 35, 12292–12302 10.1021/bi960861d 8823163

[13] Kurochkina, N. and Guha, U. (2013) SH3 domains: modules of protein-protein interactions. Biophys. Rev. 5, 29–39 10.1007/s12551-012-0081-z 28510178 PMC5418429

[14] Pandey, R. and Rodriguez, G.M. (2014) Ider is required for iron homeostasis and virulence in *Mycobacterium tuberculosis*. Mol. Microbiol. 91, 98–109 10.1111/mmi.12441 24205844 PMC3902104

[15] Gold, B., Rodriguez, G.M., Marras, S.A.E., Pentecost, M. and Smith, I. (2001) The *Mycobacterium tuberculosis* IdeR is a dual functional regulator that controls transcription of genes involved in iron acquisition, iron storage and survival in macrophages. Mol. Microbiol. 42, 851–865 10.1046/j.1365-2958.2001.02684.x 11722747

[16] Deng, Y. and Zhang, X. (2015) Dtxr, an iron-dependent transcriptional repressor that regulates the expression of siderophore gene clusters in *Thermobifida fusca*. FEMS Microbiol. Lett. 362, 1–6 10.1093/femsle/fnu053 25673661

[17] Kurthkoti, K., Tare, P., Paitchowdhury, R., Gowthami, V.N., Garcia, M.J., Colangeli, R. et al. (2015) The mycobacterial iron-dependent regulator IdeR induces ferritin (bfrB) by alleviating Lsr2 repression. Mol. Microbiol. 98, 864–877 10.1111/mmi.13166 26268801 PMC4879814

[18] Cheng, Y., Yang, R., Lyu, M., Wang, S., Liu, X., Wen, Y. et al. (2018) Ider, a DtxR family iron response regulator, controls iron homeostasis, morphological differentiation, secondary metabolism, and the oxidative stress response in *Streptomyces avermitilis*. Appl. Environ. Microbiol. 84, e01503-18 10.1128/AEM.01503-18 30194099 PMC6210122

[19] Rodriguez, G.M., Voskuil, M.I., Gold, B., Schoolnik, G.K. and Smith, I. (2002) Ider, an essential gene in *Mycobacterium tuberculosis*: role of IdeR in iron-dependent gene expression, iron metabolism, and oxidative stress response. Infect. Immun. 70, 3371–3381 10.1128/IAI.70.7.3371-3381.2002 12065475 PMC128082

[20] Schmitt, M.P., Twiddy, E.M. and Holmes, R.K. (1992) Purification and characterization of the diphtheria toxin repressor. Proc. Natl Acad. Sci. U.S.A. 89, 7576–7580 10.1073/pnas.89.16.7576 1502169 PMC49753

[21] Schmitt, M.P. and Holmes, R.K. (1994) Cloning, sequence, and footprint analysis of two promoter/operators from *Corynebacterium diphtheriae* that are regulated by the diphtheria toxin repressor (DtxR) and iron. J. Bacteriol. 176, 1141–1149 10.1128/jb.176.4.1141-1149.1994 8106325 PMC205166

[22] Lee, J.H., Wang, T., Ault, K., Liu, J., Schmitt, M.P. and Holmes, R.K. (1997) Identification and characterization of three new promoter/operators from *Corynebacterium diphtheriae* that are regulated by the diphtheria toxin repressor (DtxR) and iron. Infect. Immun. 65, 4273–4280 10.1128/iai.65.10.4273-4280.1997 9317037 PMC175613

[23] Schmitt, M.P. (1997) Utilization of host iron sources by *Corynebacterium diphtheriae*: identification of a gene whose product is homologous to eukaryotic heme oxygenases and is required for acquisition of iron from heme and hemoglobin. J. Bacteriol. 179, 838–845 10.1128/jb.179.3.838-845.1997 9006041 PMC178768

[24] Schmitt, M.P. (1997) Transcription of the *Corynebacterium diphtheriae* hmuO gene is regulated by iron and heme. Infect. Immun. 65, 4634–4641 10.1128/iai.65.11.4634-4641.1997 9353044 PMC175665

[25] Merchant, A.T. and Spatafora, G.A. (2014) A role for the DtxR family of metalloregulators in gram-positive pathogenesis. Mol. Oral Microbiol. 29, 1–10 10.1111/omi.12039 24034418 PMC3866218

[26] Tao, X. and Murphy, J.R. (1992) Binding of the metalloregulatory protein DtxR to the diphtheria tox operator requires a divalent heavy metal ion and protects the palindromic sequence from DNase I digestion. J. Biol. Chem. 267, 21761–21764 10.1016/S0021-9258(19)36677-3 1400485

[27] Wisedchaisri, G., Holmes, R.K. and Hol, W.G.J. (2004) Crystal structure of an IdeR-DNA complex reveals a conformational change in activated IdeR for base-specific interactions. J. Mol. Biol. 342, 1155–1169 10.1016/j.jmb.2004.07.083 15351642

[28] Lee, J.H. and Holmes, R.K. (2000) Characterization of specific nucleotide substitutions in DtxR-specific operators of *Corynebacterium diphtheriae* that dramatically affect DtxR binding, operator function, and promoter strength. J. Bacteriol. 182, 432–438 10.1128/JB.182.2.432-438.2000 10629190 PMC94293

[29] Pohl, E., Holmes, R.K. and Hol, W.G.J. (1999) Crystal structure of a cobalt-activated diphtheria toxin repressor-DNA complex reveals a metal-binding SH3-like domain. J. Mol. Biol. 292, 653–667 10.1006/jmbi.1999.3073 10497029

[30] Wisedchaisri, G., Chou, C.J., Wu, M., Roach, C., Rice, A.E., Holmes, R.K. et al. (2007) Crystal structures, metal activation, and DNA-binding properties of two-domain IdeR from *Mycobacterium tuberculosis*. Biochemistry 46, 436–447 10.1021/bi0609826 17209554

[31] White, A., Ding, X., VanderSpek, J.C., Murphy, J.R. and Ringe, D. (1998) Structure of the metal-ion-activated diphtheria toxin repressor/tox operator complex. Nature 394, 502–506 10.1038/28893 9697776

[32] Chen, C.S., White, A., Love, J., Murphy, J.R. and Ringe, D. (2000) Methyl groups of thymine bases are important for nucleic acid recognition by DtxR. Biochemistry 39, 10397–10407 10.1021/bi0009284 10956029

[33] Parveen, S., Bishai, W.R. and Murphy, J.R. (2019) *Corynebacterium diphtheriae*: diphtheria toxin, the tox operon, and its regulation by Fe2+ activation of apo-DtxR. Microbiol. Spectr. 7, 1–15 10.1128/microbiolspec.gpp3-0063-2019 PMC8713076 31267892

[34] Zondervan, N.A., Van Dam, J.C.J., Schaap, P.J., Martins dos Santos, V.A.P. and and Suarez-Diez, M. (2018) Regulation of three virulence strategies of *Mycobacterium tuberculosis*: a success story. Int. J. Mol. Sci. 19, 347 10.3390/ijms19020347 29364195 PMC5855569

[35] Spiering, M.M., Ringe, D., Murphy, J.R. and Marletta, M.A. (2003) Metal stoichiometry and functional studies of the diphtheria toxin repressor. Proc. Natl Acad. Sci. U.S.A. 100, 3808–3813 10.1073/pnas.0737977100 12655054 PMC153003

[36] Dussurget, O., Timm, J., Gomez, M., Gold, B., Yu, S., Sabol, S.Z. et al. (1999) Transcriptional control of the iron-responsive fxbA gene by the mycobacterial regulator IdeR. J. Bacteriol. 181, 3402–3408 10.1128/JB.181.11.3402-3408.1999 10348851 PMC93806

[37] Schmitt, M.P. and Holmes, R.K. (1993) Analysis of diphtheria toxin repressor-operator interactions and characterization of a mutant repressor with decreased binding activity for divalent metals. Mol. Microbiol. 9, 173–181 10.1111/j.1365-2958.1993.tb01679.x 8412663

[38] Guedon, E. and Helmann, J.D. (2003) Origins of metal ion selectivity in the DtxR/MntR family of metalloregulators. Mol. Microbiol. 48, 495–506 10.1046/j.1365-2958.2003.03445.x 12675807

[39] Foster, A.W., Young, T.R., Chivers, P.T. and Robinson, N.J. (2022) Protein metalation in biology. Curr. Opin. Chem. Biol. 66, 102095 10.1016/j.cbpa.2021.102095 34763208 PMC8867077

[40] Dudev, T. and Lim, C. (2014) Competition among metal ions for protein binding sites: determinants of metal ion selectivity in proteins. Chem. Rev. 114, 538–556 10.1021/cr4004665 24040963

[41] Osman, D., Martini, M.A., Foster, A.W., Chen, J., Scott, A.J.P., Morton, R.J. et al. (2019) Bacterial sensors define intracellular free energies for correct enzyme metalation. Nat. Chem. Biol. 15, 241–249 10.1038/s41589-018-0211-4 30692683 PMC6420079

[42] Osman, D., Foster, A.W., Chen, J., Svedaite, K., Steed, J.W., Lurie-Luke, E. et al. (2017) Fine control of metal concentrations is necessary for cells to discern zinc from cobalt. Nat. Commun. 8, 1884. 10.1038/s41467-017-02085-z 29192165 PMC5709419

[43] Ding, X., Zeng, H., Schiering, N., Ringe, D. and Murphy, J.R. (1996) Identification of the primary metal ion-activation sites of the diphtheria tox repressor by X-ray crystallography and site-directed mutational analysis. Nat. Struct. Biol. 3, 382–387 10.1038/nsb0496-382 8599765

[44] Rangachari, V., Marin, V., Bienkiewicz, E.A., Semavina, M., Guerrero, L., Love, J.F. et al. (2005) Sequence of ligand binding and structure change in the diphtheria toxin repressor upon activation by divalent transition metals. Biochemistry 44, 5672–5682 10.1021/bi047825w 15823025

[45] Feese, M.D., Ingason, B.P., Goranson-Siekierke, J., Holmes, R.K. and Hol, W.G.J. (2001) Crystal structure of the iron-dependent regulator from *Mycobacterium tuberculosis* at 2.0-Å resolution reveals the Src homology domain 3-like fold and metal binding function of the third domain. J. Biol. Chem. 276, 5959–5966 10.1074/jbc.M007531200 11053439

[46] Dokmanić, I., Šikić, M. and Tomić, S. (2008) Metals in proteins: correlation between the metal-ion type, coordination number and the amino-acid residues involved in the coordination. Acta Crystallogr. D Biol. Crystallogr. 64, 257–263 10.1107/S090744490706595X 18323620

[47] Chou, C.J., Wisedchaisri, G., Monfeli, R.R., Oram, D.M., Holmes, R.K., Hol, W.G. et al. (2004) Functional studies of the *Mycobacterium tuberculosis* iron-dependent regulator. J. Biol. Chem. 279, 53554–53561 10.1074/jbc.M407385200 15456786

[48] D'Aquino, J.A., Tetenbaum-Novatt, J., White, A., Berkovitch, F. and Ringe, D. (2005) Mechanism of metal ion activation of the diphtheria toxin repressor DtxR. Proc. Natl Acad. Sci. U.S.A. 102, 18408–18413 10.1073/pnas.0500908102 16352732 PMC1317899

[49] Love, J.F., VanderSpek, J.C., Marin, V., Guerrero, L., Logan, T.M. and Murphy, J.R. (2004) Genetic and biophysical studies of diphtheria toxin repressor (DtxR) and the hyperactive mutant DtxR(E175K) support a multistep model of activation. Proc. Natl Acad. Sci. U.S.A. 101, 2506–2511 10.1073/pnas.0303794101 14983039 PMC356980

[50] Stapleton, B., Walker, L.R. and Logan, T.M. (2013) Zn(II) stimulation of Fe(II)-activated repression in the iron-dependent repressor from *Mycobacterium tuberculosis*. Biochemistry 52, 1927–1938 10.1021/bi301608p 23432191

[51] Irving, H. and Williams, R.J.P. (1953) The stability of transition-metal complexes. J. Chem. Soc. 3192–3210 10.1039/JR9530003192

[52] Young, T.R., Martini, M.A., Foster, A.W., Glasfeld, A., Osman, D., Morton, R.J. et al. (2021) Calculating metalation in cells reveals CobW acquires CoII for vitamin B12 biosynthesis while related proteins prefer ZnII. Nat. Commun. 12, 1195 10.1038/s41467-021-21479-8 33608553 PMC7895991

[53] Foster, A.W., Clough, S.E., Aki, Z., Young, T.R., Clarke, A.R. and Robinson, N.J. (2022) Metalation calculators for *E. coli* strain JM109 (DE3): aerobic, anaerobic, and hydrogen peroxide exposed cells cultured in LB media. Metallomics 14, 58 10.1093/MTOMCS/MFAC058 PMC9434800 35933161

[54] Semavina, M., Beckett, D. and Logan, T.M. (2006) Metal-linked dimerization in the iron-dependent regulator from *Mycobacterium tuberculosis*. Biochemistry 45, 12480–12490 10.1021/bi060797s 17029403

[55] Tao, X., Zeng, H.Y. and Murphy, J.R. (1995) Transition metal ion activation of DNA binding by the diphtheria tox repressor requires the formation of stable homodimers. Proc. Natl Acad. Sci. U.S.A. 92, 6803–6807 10.1073/pnas.92.15.6803 7624323 PMC41417

[56] Schiering, N., Tao, X., Zeng, H., Murphy, J.R., Petsko, G.A. and Ringe, D. (1995) Structures of the apo- and the metal ion-activated forms of the diphtheria tox repressor from *Corynebacterium diphtheriae*. Proc. Natl Acad. Sci. U.S.A. 92, 9843–9850 10.1073/pnas.92.21.9843 7568230 PMC40899

[57] Pohl, E., Holmes, R.K. and Hol, W.G.J. (1998) Motion of the DNA-binding domain with respect to the core of the diphtheria toxin repressor (DtxR) revealed in the crystal structures of apo- and holo-DtxR. J. Biol. Chem. 273, 22420–22427 10.1074/jbc.273.35.22420 9712865

[58] Twigg, P.D., Parthasarathy, G., Guerrero, L., Logan, T.M. and Caspar, D.L.D. (2001) Disordered to ordered folding in the regulation of diphtheria toxin repressor activity. Proc. Natl Acad. Sci. U.S.A. 98, 11259–11264 10.1073/pnas.191354798 11572979 PMC58717

[59] Ghosh, S., Chandra, N. and Vishveshwara, S. (2015) Mechanism of iron-dependent repressor (IdeR) activation and DNA binding: a molecular dynamics and protein structure network study. PLoS Comput. Biol. 11, e1004500 10.1371/journal.pcbi.1004500 26699663 PMC4689551

[60] Wylie, G.P., Rangachari, V., Bienkiewicz, E.A., Marin, V., Bhattacharya, N., Love, J.F. et al. (2005) Prolylpeptide binding by the prokaryotic SH3-like domain of the diphtheria toxin repressor: a regulatory switch. Biochemistry 44, 40–51 10.1021/bi048035p 15628844

[61] Liu, C., Mao, K., Zhang, M., Sun, Z., Hong, W., Li, C. et al. (2008) The SH3-like domain switches its interaction partners to modulate the repression activity of mycobacterial iron-dependent transcription regulator in response to metal ion fluctuations. J. Biol. Chem. 283, 2439–2453 10.1074/jbc.M706580200 18055464

[62] Rohs, R., West, S.M., Sosinsky, A., Liu, P., Mann, R.S. and Honig, B. (2009) The role of DNA shape in protein-DNA recognition. Nature 461, 1248–1253 10.1038/nature08473 19865164 PMC2793086

[63] Rohs, R., Jin, X., West, S.M., Joshi, R., Honig, B. and Mann, R.S. (2010) Origins of specificity in protein-DNA recognition. Annu. Rev. Biochem. 79, 233–269 10.1146/annurev-biochem-060408-091030 20334529 PMC3285485

[64] Rohilla, A., Khare, G. and Tyagi, A.K. (2017) Virtual screening, pharmacophore development and structure based similarity search to identify inhibitors against IdeR, a transcription factor of *Mycobacterium tuberculosis*. Sci. Rep. 7, 4653 10.1038/s41598-017-04748-9 28680150 PMC5498548

[65] Kwofie, S.K., Enninful, K.S., Yussif, J.A., Asante, L.A., Adjei, M., Kan-Dapaah, K. et al. (2019) Molecular informatics studies of the iron-dependent regulator (ideR) reveal potential novel anti-*Mycobacterium ulcerans* natural product-derived compounds. Molecules 24, 2299 10.3390/molecules24122299 31234337 PMC6631925

[66] Yang, L., Hu, X., Chai, X., Ye, Q., Pang, J., Li, D. et al. (2022) Opportunities for overcoming tuberculosis: emerging targets and their inhibitors. Drug Discov. Today 27, 326–336 10.1016/j.drudis.2021.09.003 34537334

[67] Rodriguez, G.M., Sharma, N., Biswas, A. and Sharma, N. (2022) The iron response of mycobacterium tuberculosis and its implications for tuberculosis pathogenesis and novel therapeutics. Front. Cell. Infect. Microbiol. 12, 876667 10.3389/fcimb.2022.876667 35646739 PMC9132128

[68] van Bergeijk, D.A., Terlouw, B.R., Medema, M.H. and van Wezel, G.P. (2020) Ecology and genomics of actinobacteria: new concepts for natural product discovery. Nat. Rev. Microbiol. 18, 546–558 10.1038/s41579-020-0379-y 32483324

[69] Hutchings, M., Truman, A. and Wilkinson, B. (2019) Antibiotics: past, present and future. Curr. Opin. Microbiol. 51, 72–80 10.1016/j.mib.2019.10.008 31733401

[70] Young, T.R. and Xiao, Z. (2021) Principles and practice of determining metal-protein affinities. Biochem. J. 478, 1085–1116 10.1042/BCJ20200838 33710331 PMC7959690

